# The NCCB Case Example: Reflections on a Successful Fourteen-Year CBPR Partnership

**DOI:** 10.35844/001c.120896

**Published:** 2024-07-29

**Authors:** Alexis D. Jemal, Ellen Benoit, Shola Thompson, Heather A. Jones, Liliane Windsor, Teri Lassiter, Warren Thompson, The NJ Critical Consciousness Collaborative Board (3CB)

**Affiliations:** 1 Silberman School of Social Work, Hunter College; 2 The Briar Patch Collaboratory; 3 North Jersey Community Research Initiative; 4 Social Welfare, The Graduate Center, CUNY; 5 School of Social Work, University of Illinois Urbana-Champaign; 6 School of Public Health, Rutgers, The State University of New Jersey; 7 Newark School of Arts and Sciences, Rutgers, The State University of New Jersey; 8 Lincoln Park Coast Cultural District

**Keywords:** CBPR, critical consciousness, community-engaged research, social determinants of health, capacity-building, collaboration, advocacy

## Abstract

The Newark Community Collaborative Board (NCCB) is in its fourteenth year of operation with nine successful research projects and more than six million dollars in funding. The NCCB began with a community needs assessment in Newark, New Jersey, that led to the subsequent establishment of a community collaborative board (CCB) of consumers, researchers, service providers, and residents committed to advocating for health equity through community engagement and research informed by critical thinking. This paper explores the NCCB’s history and processes that allowed conducting community-based participatory research (CBPR) to reduce inequities related to social determinants of health (SDH). This conceptual manuscript draws on data from NCCB meeting minutes and a group interview with three of the five founding members. We detail the collaborative process used to develop and assess Community Wise, a multilevel, group-based intervention designed to reduce substance use among formerly incarcerated men in Newark, funded by the National Institutes of Health. Review of documentation and interview transcripts revealed the following key ingredients for success: 1) Having a north star; 2) Functional diversity; 3) Challenges as learning opportunities; 4) Board structure and healthy relationships; and 5) Funding and resources. The NCCB has undergone multiple transformations, including a name change to the New Jersey Critical Consciousness Collaborative Board (NJ-3CB), representing its growth from being a small local board to becoming part of a network of community collaborative boards across the United States and a chapter of the global campaign against racism. These and future transitions will help sustain the collaborative journey.

Established in 2010, the Newark Community Collaborative Board (NCCB) — a group of community-based practitioners, consumers, community members, and academic researchers — integrates interdisciplinary and participatory methods in conducting community-engaged, action-oriented research to reduce structural racism and make progress toward health equity. The NCCB is an example of a community-academic partnership that engages in community-based participatory research (CBPR), participatory action research, and community-engaged research. For example, the NCCB’s collaborative process led to the development and testing of *Community Wise*, a multilevel intervention designed to reduce substance use among formerly incarcerated men in Newark, New Jersey. Committed to championing health equity through community engagement, critical consciousness development, and rigorous research, since its creation the NCCB has achieved significant milestones, successfully taking on nine research projects and securing more than six million dollars in private and federal funding. Utilizing data from NCCB meeting minutes and interviews with three of the five still-active founding members, this paper details how and why the NCCB came into existence, its processes for successful collaboration (including lessons learned from challenging experiences), and thoughts about future directions on the collaborative journey of CBPR (Drahota et al., 2016) to address inequities related to social determinants of health (SDH).

Structural racism is a major SDH. A substantial body of research documents the impact of structural racism compounding the significantly higher disease burden carried by marginalized people in the U.S. Whether as a result of unequal access to medical care or implicit bias among providers, Black adults tend to receive diagnoses later for a number of progressive diseases, including HIV (Chapin-Bardales et al., 2017) and cancer (Farias et al., 2020), and to be less likely than Whites to be prescribed adequate medication for pain (Hoffman et al., 2016; Odonkor et al., 2021). Low-income communities of color are disproportionately located near toxic facilities that are linked to illness (Burbank et al., 2023; Howarth & Eiser, 2023; Payne-Sturges et al., 2023; Shrestha et al., 2023). Despite rates of substance use similar to those among Whites (Center for Behavioral Health Statistics and Quality, 2021), Black/African Americans are disproportionately incarcerated for drug-related offenses, a consequence of selective enforcement of drug-control laws. Black/African Americans are also more likely to experience overdose (Larochelle et al., 2021) and unequal access to high-quality treatment (American Society of Addiction Medicine, Inc., 2021; Farahmand et al., 2020).

Although the harms of structural discrimination are well documented, research is lacking on strategies to combat racism, increase trust among government, community, and scholars, and develop evidence-based interventions to address disproportionate health consequences. Recently, scholars and government agencies have been calling for new solutions to combat racism and address the disproportionate impact that SDH have on marginalized communities (Adler et al., 2016; Weerasinghe & Tawa, 2021). Increasingly, the recommended approach to developing such solutions has included engaging communities in intervention development. Universities have started to acknowledge the value and rigor of community-engaged research as a way to increase the real-world impact of scholarship and address systemic racism in research (Wallerstein et al., 2018). Moreover, the National Institutes of Health (NIH) encourages community-engaged scholarship on many of its funding opportunity announcements by requiring community partners’ participation.

Community-Based Participatory Research (CBPR) is a collaborative, community-engaged approach to scholarship that empowers community members to define problems that affect them and to shape solutions (Israel et al., 2005; Wallerstein et al., 2018), thereby combatting historic mistrust of researchers. It integrates methods of scientific inquiry with community capacity-building strategies to improve health and address community needs (L. C. Windsor, Pinto, et al., 2014). CBPR integrates academic and experiential knowledge in the research process under a formal structure in which non-research community members are equal partners with scientists. The relationship is influenced by Freire’s (2000) work on critical consciousness and praxis, establishing the idea that community and academic partners learn from each other in a non-hierarchical capacity. This way, the researchers are not the only experts as community members shift from being passive objects of study to participating in the inquiry (Wallerstein et al., 2018). Thus, community-academic partnerships, community inclusion, and public engagement are woven into all phases of the research. Levels of community engagement vary along a continuum in which the highest level is found in CBPR (Goodman & Sanders Thompson, 2017).

This shifts the research process from a positivist orientation, in which knowledge includes only that which can be measured, to one that encompasses interpretation (e.g., mixed quantitative and qualitative research). In other words, it checks the hegemony of the ivory tower with indigenous expertise (Israel et al., 1998; Wallerstein & Duran, 2018). Because research conducted within a CBPR framework blends experiential and scientific knowledge, it is considered promising for the development of sustainable interventions to reduce inequities caused by social determinants of health. As such, CBPR has been used in a number of ways to address community health (e.g., Bogart & Uyeda, 2009), violence prevention (e.g., Oscós-Sánchez et al., 2021), and environmental concerns (e.g., Cook, 2008; Palaniappan, 2004). CBPR has been criticized as too slow and not constituting valid scholarship, but these concerns often reflect institutional elitism (Blumenthal, 2011; Calleson et al., 2005) grounded in a structurally racist knowledge system favoring positivism (Ladson-Billings, 2020; Wallerstein & Duran, 2018; Zuberi & Bonilla-Silva, 2008). The NCCB’s experience invites a reevaluation of these criticisms, positioning CBPR as a transformative and inclusive research paradigm that values multiple ways of knowing and actively engages communities in the research process.

CBPR may improve research rigor and outcomes (Drahota et al., 2016; Wallerstein et al., 2020). For example, involving community members from the beginning of a project may improve participant recruitment and increase retention by capitalizing on trusted relationships (Brush et al., 2020; Muhammad et al., 2015). Community input can improve the feasibility of methods used by clarifying questions in interview and survey instruments, leading to better quality data (Muhammad et al., 2015). Community involvement can also contribute to a project’s sustainability by helping to ensure that the intervention is acceptable to the local population and that multiple parties have vested interests in supporting the program (Israel et al., 2018; Wallerstein et al., 2018). Finally, building community capacity for research and program implementation may encourage the renewal of funding and other support, which also contribute to sustainability (L. C. Windsor et al., 2021). The implementation of CBPR projects has generated a need for Community Collaborative Boards (CCBs) whose members are diverse (e.g., academics, service providers, community members) and who work collaboratively. The benefits of CBPR notwithstanding, there are several challenges to engaging in CBPR methods, including power sharing, decision making, institutional biases, and lack of capacity (e.g., skills, time) and resources (e.g., staff, space, funding) (Garzón et al., 2013; Minkler, 2005; Wallerstein et al., 2019; L. C. Windsor et al., 2024).

Although CBPR has been around for more than 20 years, the literature has limited accounts of CBPR applications in real world settings where researchers and community partners negotiate scientific rigor, funding sources, community needs, differences in perspectives, and the administrative challenges of conducting rigorous and innovative research informed by CBPR principles. In this paper, we use a case example to discuss challenges and strategies in implementing CBPR principles to develop and assess an intervention to reduce substance use among formerly incarcerated men in Essex County, New Jersey.

This case example is based on archival records and a group interview, qualitative methods suited for understanding social processes by answering “how” and “why” questions (Tenny et al., 2024). The NCCB maintains an extensive online library in the form of a secure Box account at the University of Illinois. The archive contains material documenting the NCCB’s history, from the initial community needs assessment and formation of the original board in 2010 to present work as the reimagined Critical Consciousness Collaborative Broad (3CB). The library contains thousands of documents, including agendas and minutes from NCCB committee and full-board meetings since 2010, grant proposals, published and unpublished manuscripts, training materials, evaluations, and correspondence. The archive also includes audio and video recordings of intervention sessions, selected committee meetings, and training activities. For this article on the methods, strategies, and processes of the NCCB for engaging in participatory research, we primarily relied on meeting minutes and our published articles as documentary sources. Although the NCCB archive includes minutes, notes, and agendas from more than 130 meetings, we selected those from key time periods in NCCB history. We read minutes mainly to accurately document the chronology of events and to reconstruct the development of relationships among board members and the decision-making processes they employed in seeking funding, negotiating priorities, and creating the *Community Wise* intervention.

The story of the NCCB engaging in CBPR is also heavily informed by insights from a group interview that the first author conducted with three of the NCCB’s founding members, who remain active on the board. They recalled their motivations for getting involved, their evolving expectations regarding the board’s purpose and their assessments of how the NCCB manages growing pains and other challenges. The interviews were recorded, transcribed, and reviewed to identify the NCCB’s foundational principles and to provide context and meaning for the documentary information. This triangulation of data sources (Farmer et al., 2006) reveals the NCCB’s history and details its current reality and foreseeable future.

## The NCCB as a Case Example

The history of the NCCB highlights how community members and researchers collaborated within the CBPR paradigm to develop and assess an intervention addressing substance use among formerly incarcerated men. The story of the NCCB illustrates how members of the community and the academy bring differing perspectives to a shared mission to create change. The process includes confronting material and social obstacles to establish trust and build a cohesive organization. We highlight some of the lessons learned by the NCCB as it overcame challenges to achieve successful collaboration, demonstrating the critical importance of community voices to the transformative potential of CBPR in addressing structural health inequities.

### History of Collaboration

The NCCB was developed as part of the research agenda of Dr. Liliane Windsor (“Lili”), who came to the U.S. from Brazil to complete her doctoral studies at the University of Texas-Austin. After defending her dissertation, Lili joined the faculty in the School of Social Work at Rutgers University in Newark, NJ. During her dissertation work, Lili became aware of a “disconnect between the typical treatment service and the experiences of Black communities living in poverty” (L. Windsor, personal communication, 3/5/2023), which transformed the way she engaged in substance use disorder treatment research. After further considering the impact of macro-level social forces on substance use, Lili decided to develop and test a multi-level intervention informed by critical consciousness. Consequently, Lili turned to feminist perspectives and Paulo Freire’s critical consciousness models as theoretical frameworks to provide alternative perspectives and ways of examining substance use and treatment.

Paulo Freire, a Brazilian educator who is considered one of the most brilliant educational thinkers of the late 20th century, defined critical consciousness as the ability to “perceive social, political, and economic contradictions, and to take action against the oppressive elements of reality” (Freire, 2000, p. 19). Critical consciousness theory (CCT) argues that oppressed groups, such as individuals residing in predominantly low-income and African American neighborhoods with a history of substance use disorders and incarceration, are disempowered and dehumanized through objectification and silencing. Yet these individuals also possess strength in the form of knowledge and skills. When they join other oppressed individuals in dialogue, critical consciousness develops and entire groups of people become empowered to create individual and community change (Freire, 2000; Mullaly & West, 2018; L. C. Windsor, Jemal, et al., 2014; L. C. Windsor, Pinto, et al., 2014). When applied to behavioral health interventions, this framework conceptualizes substance use as a form of internalized oppression and self-care (reduced substance use) as an act of resistance.

During this time Lili learned about CBPR from Rogério Pinto, a fellow Brazilian, who had organized a community collaborative board (CCB) in New York. In keeping with CBPR principles, Rogério’s approach focused on establishing a community board that was truly collaborative, rather than advisory as in traditional research. Members were recruited from varied professional and community networks and educated each other about their networks and resources at board meetings. They also recorded several meetings in order to understand group dynamics and ensure equitable participation (Pinto et al., 2011). Lili adapted this approach to the Newark, New Jersey context.

Armed with a theoretical model for how to conceptualize problems and a model for process or ways to do the research (CBPR), Lili began sharing her innovative ideas with like-minded people in academia and community-based organizations (CBOs) providing services in Newark. She began with a small project in partnership with the North Jersey Community Research Initiative (NJCRI), a CBO that had been providing health and social services in Newark for decades. With a small grant ($5,000) from the Rutgers Center on Behavioral Health Services and Criminal Justice Research (the Center), Lili worked in partnership with NJCRI on a concept mapping project using co-developed research questions, which served as a needs assessment of Newark, to provide a foundation for the future CBPR work. Participants included 75 community members, alcohol and substance users, service providers and researchers (L. C. Windsor, 2013; L. C. Windsor & Murugan, 2012).

**Figure 1.**
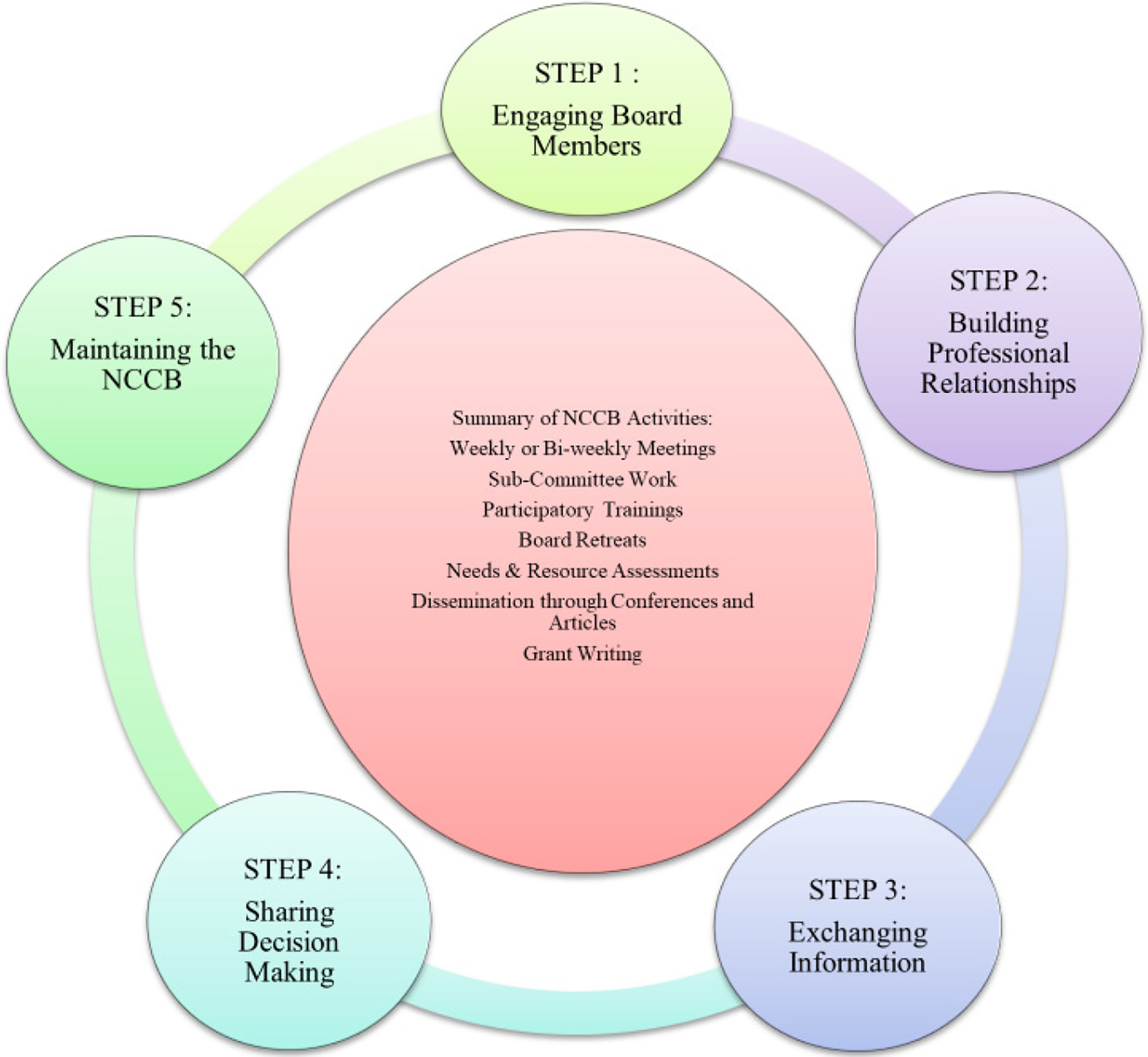
Newark Community Collaborative Board framework Windsor, et al. (2014). *Community Wise:* Development of a model to address oppression to promote individual and community health. *Journal of Social Work Practice in the Addictions, 14*(4), 402–420.

This project allowed Lili to familiarize herself with Newark and to talk with people, learn what they thought about the role of drugs and alcohol in the community, and develop working relationships. After the needs assessment was completed, Lili sent an email to community leaders, researchers, community members, and consumers to explain the concept of CBPR and the project idea, and invited them to become members of a new community collaborative board. Those who expressed interest were invited to a mixer that also included a presentation on critical consciousness theory and CBPR. As an innovative way to facilitate mixing and mingling, Lili and the team asked the attendees to interview and estimate each other’s level of interest using suggested questions related to critical consciousness and the project goals. For example, attendees were asked about their connection to Newark. People with the strongest recommendations were invited to become CCB members while others were invited to attend as guests with the option of becoming a member later (L. C. Windsor, Pinto, et al., 2014). In addition to the mixer attendees, other people in various community positions were invited to apply, yielding an initial group of approximately 30 people. Figure 1 illustrates the framework that informed the creation of the NCCB.

Two original members of the NCCB talked about why they joined the board. Dr. Teri Lassiter, one of our co-authors and original NCCB member, recalled that she was doing other community work in Newark at the time and the invitation seemed like a great way to get to know and connect with different groups of people. Since then, she has found these connections and the board work have led to other opportunities to engage with coalitions aligned with her research interests. Warren Thompson, another co-author and original NCCB member, was working within the field of prisoner reentry as Newark was beginning to see the backlash of the mass incarceration movement. High numbers of individuals were returning to Newark communities after periods of incarceration. Because of his lived experience with the Newark community, Thompson was invited to the mixer. Once Warren learned more about the approach, he wasenthralled. Being born and raised in Newark and working as a community worker and service provider, Warren witnessed firsthand the oppression that many Newark families experienced. As such, he valued approaching service provision from a systemic perspective.

## The First Year: Board Development

The NCCB had its first meeting on October 4, 2010, in a conference room at Rutgers University. Thirteen members and guests were present, representing service consumers, community members, academics, and other stakeholders. For two hours the group shared thoughts on the purpose of the board and set up a preliminary schedule of future weekly meetings and activities (Newark Community Collaborative Board, 2010). The first few meetings focused on determining the NCCB’s goals. Attendees explored topics they cared about in the City of Newark and agreed to focus on substance use, HIV prevention, and criminal justice. The conversation at one point centered on working with youth, but the NCCB decided to focus on adults.

One of the first tasks for the NCCB was to develop bylaws that governed how the group would operate. At the initial meeting, the board drafted a mission statement and created a procedures and governance committee to finalize the bylaws following Rogério’s model. In addition to the mission statement, the bylaws stated the NCCB’s vision and goals, described the board’s structure and delineated self-governance procedures (e.g., membership, attendance and voting rights, and duties). To ensure equitable participation and input from all board members, the bylaws were designed as a living document and were revised several times to incorporate ideas and perspectives that emerged from group meetings (NCCB, 2010). The NCCB’s vision is to build healthy communities where members freely exercise their civil rights and advocate for equitable opportunities for all. The mission is to partner with marginalized communities to promote health equity through critical consciousness building in research, community engagement, critical dialogue, and civic participation.

To govern meetings, the NCCB initially adopted Roberts’ Rules of Order but eventually elected fewer formal guidelines. Similarly, the NCCB’s bylaws have undergone numerous revisions regarding attendance and procedures over the years, following the preferences of board members, and had been largely done away with by spring of 2022 (NCCB, 2022). This reflects the NCCB’s maturity into a stable group operating on trust that any member who wanted to leave or make changes would simply speak up. It also accommodates the board’s evolution into part of the Critical Consciousness Collaborative (3C), which includes collaborative boards in New York, Illinois, and Michigan and necessitates virtual meetings. However, the NCCB’s description, vision, and mission have remained intact, adapted by 3C (http://www.the3c.org/about-us.html). Although the NCCB’s structure has evolved organically, it continues to describe itself as a collaborative board of diverse community members (e.g., those affected by health inequities, researchers, service providers, and government representatives) grounded in critical consciousness and working together to build safer and healthier communities through engagement in CBPR, service, and advocacy.

The NCCB met weekly for most of the first year, focusing on building relationships, determining how the NCCB would work together, the focus of the work, and establishing a community of learning. “The first year was essential,” according to Warren, a foundational year that is credited with building-in sustainability. In keeping with CBPR principles, board members trained each other in relevant skills and areas of expertise. “The first year of the NCCB was all about training and dialogue so we could be on the same page,” Lili recalled. “We were all learning together and we were building the NCCB together. So, we spent about a whole year together, just learning and processing that, learning to figure out what the heck we are doing.”

The board set up a calendar on which members volunteered to share their expertise, sometimes with PowerPoint slides and sometimes as unaided talks. Lili continued:

So, we had training on funding. How do you pursue funding and do grant writing? How do you write a grant research methods section? How do you develop an intervention? Because we knew we wanted to develop an intervention. We had trainings on the science of substance use treatment. What are the different options? The people who were leading these trainings worked in combinations.

Lili explains that the training on criminal justice paired a researcher and a consumer of substance use treatment and reentry services. The researcher provided information on criminal justice research, and the consumer-partner provided context informed by his personal and lived experience. Lili paired with an administrator of a CBO that provided substance use treatment and sexual health services. Lili provided the science, and the administrator-partner offered local experience from the perspective of a service provider in Newark. In addition to these training pairs composed of board members, there were also trainings about Newark, New Jersey, offered by community members. These presentations at NCCB meetings discussed the unique experiences that occurred in Newark, such as navigating re-entry processes and engaging in community action. Community partners were compensated for their time and food was offered at each meeting.

Through Lili’s study of critical consciousness as a theoretical framework, she learned about the Paulo Freire Institute at UCLA and invited the director and an affiliated popular educator to provide some training for the NCCB. They flew to Newark and engaged the board in a day-long retreat focused on understanding the principles and methods of critical consciousness theory. The training included didactic instruction and group discussions, and NCCB members completed pre- and post-tests measuring their knowledge of critical consciousness. The experts gave the board copies of the instructional materials to keep for refresher training and to train new members (NCCB, 2010).

In addition to formal or organized training sessions, there was a consistent exchange of resources and information about what was happening on the streets and at local agencies. For example, every meeting began with updates from board members. Teri Lassiter summarized the importance of the learning process by noting that the group had numerous conversations on many topics. They had assigned reading materials and there were guest speakers. In addition to learning, these meetings also served as a weeding out process (i.e., people who were less committed voluntarily left the board) and facilitated investment in the work. After attending a few meetings, people gained a clear idea of what the work was about and could make informed decisions about whether to continue with the NCCB. Teri noted, “It wasn’t just like we’re gonna sit down, eat, drink soda, and hang out and chit chat. We’re actually sitting here, going through a process of understanding.”

Similarly, Warren observed that they were learning new concepts and trying to absorb a lot of information while trying to become familiar with new people: “There were rotating people, but there was this core group that showed up all the time.” However, Teri added, “the meetings were always a little bit different depending on who attended. Everybody really contributed to the conversations that we had, which was important.” Teri noted that some participants’ contributions took the meeting in a different direction than planned, but no one was ignored, and everybody’s opinion could be discussed, “whether you agreed with them or not. I think it’s important when you’re listening to the community members that you make sure that their voices are heard just like the researchers’ perspectives. You know we’re all on the same board.”

In the spring of 2011, the NCCB received a $75,000 grant from the Center to conduct an ethnographic and photovoice study to understand the lived experiences of individuals transitioning from incarceration into communities in Newark. The team conducted focus groups, in-depth individual interviews, and field observations with 28 individuals, including formerly incarcerated people, family, friends, and service providers. The project culminated in an exhibition of photos taken and annotated by the formerly incarcerated participants, held at the Rutgers University Campus Center in Newark at the beginning of 2012.

## Years 2 to 12: Projects and Accomplishments

After the board was established and in full working mode, with some work delegated to committees, full board meetings happened monthly instead of weekly. Yet they still generally took place in the early evening and lasted for two hours (usually 6–8 p.m.). Meeting times and days were determined by group consensus; virtual attendance by video was an option beginning with Meeting #2 (NCCB, 2010). By 2012, the NCCB had stabilized as a 16-member group that was diverse in terms of age and racial and gender identity, as seen in Table 1 (L. C. Windsor, Pinto, et al., 2014). Members were adults involved in community action and interested in critical consciousness and improving support for formerly incarcerated people struggling with substance use.

The NCCB was also using technology, including file-sharing platforms, to build a library of resources, including archived agendas and minutes from all meetings (Jessell et al., 2016). This use of technology not only facilitated participation, but encouraged accountability. “We evaluated everything we did, and we documented everything that we did from day one,” Lili recalled. For much of the NCCB’s history, members submitted membership renewal applications every two years and completed leadership evaluation forms every year. Evaluation of the NCCB’s processes were an integral component of the NCCB’s praxis and collective critical consciousness development. The NCCB also published an article on its use of technology (Jessell et al., 2016) and another detailing the process by which it evaluated the feasibility of the pilot version of *Community Wise* (L. C. Windsor, Jessell, et al., 2014). These early publications reported on the work of NCCB committees developing the manual content and on some members serving as group facilitators. Pilot data was analyzed by the academic partners, who took the lead on writing the manuscripts after reviewing results with the board. It should be noted that all NCCB publications invited community partners to participate. The community partners that had the interest and capacity to do so became co-authors and were credited for their contributions.

The NCCB started developing the intervention and scales to assess its impact at the end of the first year. At this point, the NCCB submitted their first grant application to the National Institute of Minority Health and Health Disparities (NIMHD), to develop and test *Community Wise*. Although the NCCB received an outstanding score from the scientific peer review, they weren’t funded. As a result, the Center (P30) again funded the NCCB with $260,000 in August 2011 to develop and pilot the *Community Wise* intervention. The board formed committees that developed the intervention content and collaboratively drafted the facilitator and participant manuals. All committee work was reviewed and approved by the full board. For example, one committee identified topics related to oppression that would prompt critical reflection during group intervention sessions. The board approved topics and commissioned an artist to paint images representing each issue so that intervention participants could launch critical dialogue by reacting to the illustrations (NCCB, 2011).

**Figure 2.**
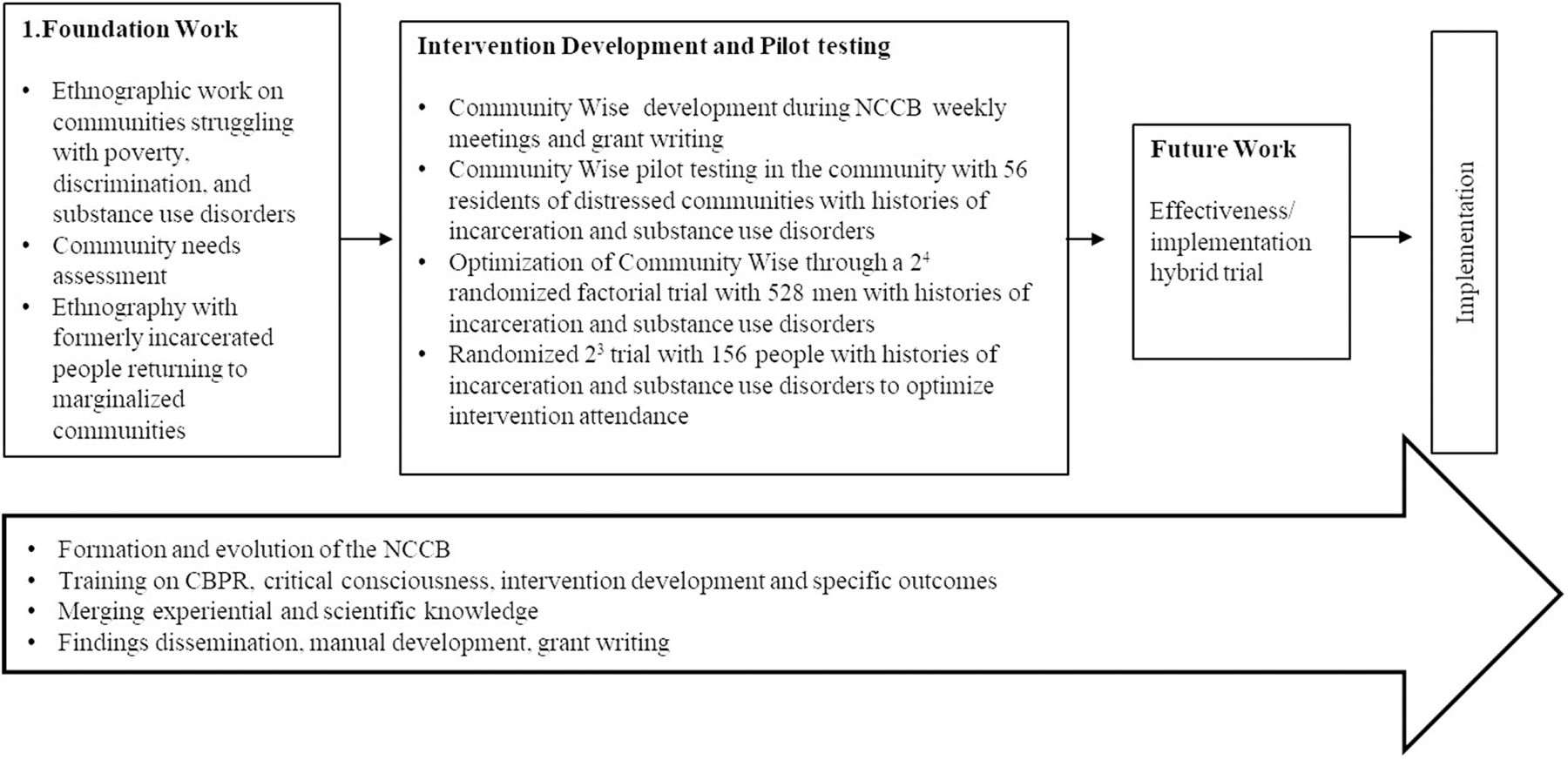
Research Agenda Snapshot

The pilot study gave the NCCB a foundation for exploring if *Community Wise* produced the hypothesized outcomes. In early 2015, the board established a Theoretical Committee comprised of seven board members who drafted a survey instrument to measure critical consciousness and parameters for capacity-building projects as a critical action component of the intervention (NCCB, 2015). With experience writing a federal grant application, the NCCB revised *Community Wise* for optimization and successfully applied for funding from NIMHD to do the optimization study at a CBO. Figure 2 provides a schematic representation of the NCCB’s research agenda in developing and testing *Community Wise*.

*Community Wise* is a manualized, multi-level behavioral intervention grounded in critical consciousness theory (CCT), designed to help participants develop a deep understanding of how SDH influence health inequities related to substance use. Participants then apply this knowledge to critical action against inequities at the *micro-level* (e.g., cognitive and behavioral processes); the *meso-level* (e.g., relationships with individuals and organizations); and the *macro-level* (e.g., political and cultural processes). From a critical consciousness perspective, self-care and community engagement are means of resisting oppression. *Community Wise* participants develop critical consciousness through three components, beginning with core training in critical thinking (two sessions), learning to evaluate beliefs and question assumptions. In the critical dialogue (CD) component (six sessions), illustrations depicting several forms of oppression prompt discussions in which participants employ critical thinking to examine how SDH has affected their lives and the health of their communities. In the final component, participants identify and engage in capacity-building projects (CBP, six sessions) intended to improve conditions in their communities.

In 2023, the NCCB completed the *Community Wise* Optimization Study that utilized the multiphase optimization strategy to engineer the most efficient, effective, and scalable version of the intervention that could be delivered for $250 per person or less (L. Windsor et al., 2018). The study was designed to detect change in alcohol and illicit drug use in a sample of 528 men with histories of substance use disorder and incarceration, residing in Newark (L. Windsor et al., 2018; L. C. Windsor et al., 2021). (For study results and methods, please see publications at the3c.org.)

## Other Projects and Expansion Beyond New Jersey

The NCCB has also been involved in other projects not included in Figure 2. In 2014, board members recruited and interviewed providers from 20 social service agencies for an NIH-funded study of interagency collaboration (Project ICI) conducted by Rogério at Columbia University. Two NCCB members, both graduates of *Community Wise*, participated in educational programs at Essex County Community College in 2019 and became interested in developing a course to train service providers to incorporate critical consciousness into their practice. They received training as *Community Wise* facilitators from Lili and worked with other NCCB members to draft a proposal for funding to adapt the intervention manual to train providers in SDH and critical consciousness. Although the proposal was never completed, they did adapt the manual and train two groups of service providers at the college. This is an example of how CBPR can facilitate community capacity building.

In September 2020, the NCCB became a chapter of the Campaign Against Racism (CAR), an international network of organizations working toward health equity by dismantling structural racism (http://www.equalhealth.org/campaign-against-racism). CAR leadership offers support to help chapters achieve local objectives. For the NCCB, the goal is essentially its mission: To partner with marginalized communities to combat health inequities through research, community engagement, critical dialogue, and civic participation. In response to the devastating impact that COVID-19 had in Newark, the NCCB pivoted and received two research grants from the National Institutes of Health (NIH) to optimize adaptive interventions designed to encourage members of medically and socially vulnerable populations to test for COVID-19 and adhere to CDC and state health department prevention and treatment recommendations. The research design involved adapting two evidence-based interventions that had proven successful in HIV testing and treatment following CBPR principles. The first grant supported the research with 602 residents in Essex County (Newark), New Jersey; the second funded the addition of a study site in St. Clair County, Illinois (East St. Louis), where 554 participants were enrolled. Although the NCCB retained its original form, COVID-19 forced the group to meet virtually. Also, NIH imposed accelerated deadlines for the COVID-19 research, and we adapted existing interventions so the NCCB role became somewhat more advisory than formative. However, NCCB members did create literature on testing and vaccination for participants.

## Challenges and Lessons Learned from the NCCB Experience

Several key principles for success emerged from the NCCB’s process, which the founders/interviewees underscored as secrets to longevity that may benefit others who are interested in doing this important work. The below examples are descriptions of actual events that were discussed in board meetings (as recorded in the minutes) with context provided through the interviews with the original board members.

### Funding

The funding secured by the NCCB in the first year from the Center allowed us to compensate our board and staff members and conduct significant foundational research that positioned us well to expand our funding sources. Funding for administrative work is critical to support the research and collaborations (Tendulkar et al., 2011). Over the years, the NCCB has applied for several foundation grants to build community capacity, without success. One obstacle is the common requirement that grant recipients have 501(c)(3) status, which the NCCB lacked. The board formed a committee to investigate the feasibility of becoming a 501(c)(3) organization and discussed pros and cons at several meetings, ultimately deciding that it would not be feasible (NCCB, 2019).

Somewhat less existentially, the NCCB faced and managed several challenges implementing the large NIH grant awarded to develop and test the *Community Wise* intervention. Lili operated from a place of transparency with the board; she addressed questions and concerns regarding budget decisions and exercised capacity-building approaches with the team. Operating with mutual respect, transparency, and openness increases the successes for long-standing CBPR partnerships. Within this framework, there is an acknowledgment of power differentials that recognizes the varied priorities of the academic institution and CBO (Bush et al., 2019). For example, Lili educated staff on university protocols and financial structures. However, issues regarding funding persisted and presented barriers for the sustainability of the NCCB. First, universities often retain the largest portions of the grants, despite the CBO doing the bulk of the community work. This type of financial model created a “power discrepancy” between agencies and academic institutions. Lili explains:

One of them is that, initially, the money always went solely through the university. Later on, I was able to add our partnering CBOs as sub-awardees and flow more funds to them. I’m very proud and excited to see that shift, because now the huge bulk of the budget really goes to the community-based organizations. However, major power discrepancies remain. For instance, the university has agreements with the federal government to receive approximately 56% of the budget in indirect funding that covers overhead and administration of the grant. CBOs cannot negotiate more than 36% in indirect funds, even if they are hosting all the research activities.

NCCB researchers sought to rectify this funding challenge by urging agencies to increase their rates to ensure a fairer distribution of funds. This would have allowed agencies to see an increase in earnings from the grants. The strategy could be a critical step towards disrupting the imbalance of power as the relationship between community agency and institution is reciprocal — agencies often incorporate researchers’ funding in their budget trajectory and researchers need community partners for effective implementation of interventions.

Another challenge was delays in payment to the CBOs from the University. CBOs often performed the work with their own funds and submitted invoices to the university for reimbursement. Lili recalled receiving monthly bills as high as $70,000 from a partnering CBO. Community organizations often operate from limited funding pools and are situated in under-resourced communities. So, while the agency depended on the institution as a funding source, frontloading expenses caused a strain on the agency’s budgets. The board had conversations to remedy this challenge, and one included identifying funding that would pay the CBO directly. We also advocated with leadership at the National Institutes of Health to contract directly with CBOs to cover their share in the grant or add requirements to contracts expecting universities to either offer cash advance to CBOs or expedite the reimbursement.

Pay inequities are also reflected in the compensation of work conducted by community partners and academics, reflecting structural biases about the value of community member contributions and researchers’ contributions. Salaries are dependent on several factors, including credentials and formal education. The board prioritized hiring and recruiting members who were heavily affiliated with the community regardless of personal resources, including community stakeholders with criminal backgrounds and those who never completed high school. For professional researchers, salaries are related to the percentage of their overall time that is dedicated to funded CBPR projects, whereas other NCCB members are paid hourly for their CBPR work at much lower rates. A second part of this challenge is related to the generally low pay of employees at CBOs, which are chronically underfunded. One of the NCCB’s NIH grants included outreach and peer navigation positions at $40,000 yearly in the budget, a relatively low salary. Yet before agreeing to this, the partner CBO initially objected, saying that amount was higher than their other staff salaries and would cause internal strife among employees. The NCCB interrogated pay disparities from a critical consciousness lens and was able to elevate the recognized value of experiential knowledge in this one instance, but the struggle highlights structural barriers to achieving equity.

### Leadership

NCCB members asked Lili to chair the board and serve as the contact investigator on most of the board’s grants. However, in 2014 Lili was denied tenure at Rutgers University and moved to Illinois in 2015 after accepting a new faculty position at the University of Illinois at Urbana-Champaign. This caused major disruptions and the board had to reinvent its practices to accommodate a long-distance leadership structure in which Lili was able to continue chairing the NCCB virtually for six years. However, this transition impacted the quality of board meetings and productivity, particularly when Lili decided to focus on organizing a community collaborative board in Illinois. The NCCB struggled to identify a local chair or leadership team (NCCB, 2021). Board member Warren stated,

It was because the Board had gotten so comfortable depending on Lili, Rogério, and Ellen, that when Lili went to Illinois, it created this kind of vacuum because her time was being spent in other places. And then, even when we tried to fill that, right, or someone tried to step up to fill it, right… the Board is looking at these people like, come on, man, you gotta be kidding. Where is Lili? And you know, thinking about it in hindsight? I think that a transition of power is needed to a capable leader…

This quote illustrates that, though not officially a top-down leadership style, convenience led Lili to lead the board. Despite attempts to equally distribute power on the board and create a more equitable governing body, the board members’ interviews revealed that there was an internalization of power that identified the research professionals as the leaders. This is an ongoing struggle for the NCCB.

### Power

Research is inherently entwined with power dynamics and the board had to acknowledge past harms instigated by academia on marginalized communities at-large, such as reinforcing stigma (Sevelius et al., 2020) and reproducing power imbalances (McCracken, 2020; Wallerstein et al., 2019). To overcome gaps in power and privilege (Garzón et al., 2013; Muhammad et al., 2015; Wallerstein et al., 2019), the board engaged in critical dialogue regarding such topics as power dynamics, hierarchies, authority, credentials, social identities, and transparency, especially pertaining to money and budget. Such topics were inherent in the NCCB’s grounding in critical consciousness theory and are summarized in many, if not most, of the meeting minutes, particularly when issues of board structure or developing and evaluating the intervention were on the agenda.

These conversations delved into the nuanced power relations between the CBOs and the board (including the dual roles that some members have as they serve as staff at the CBO) and the university and the CBO/board. With three distinct entities striving to coexist, each including individuals with varying proximity to the research interests, navigating these relationships became complex. Issues include determining who “owns” or will control data and intellectual property rights, trust/mistrust, equity/inequity of resources, and who benefits from the research. Challenges range from the practical to the personal. For example, the demands of academic research (e.g., randomization and inclusion of a control group) may conflict with the community’s ideals or needs (Ross et al., 2010). Careful planning, open communication, and shared decision making are essential to combating these practical obstacles. Personal issues may include building trust, ensuring effective communication (Strickland, 2006), and sustaining relationships, morale, and commitment to the CBPR process over time (Israel et al., 2018). Challenges to these personal connections include lack of time, resources, interrupted funding, and inequities among partners in receiving benefits. The departure of original members, addition of new members, and/or active members missing numerous meetings can affect the partnership’s identity and focus (Israel et al., 2018).

### Trolling and threats of violence

In a polarized environment where any issue can be weaponized to advance political agendas, conducting CBPR to promote health equity poses unique challenges, especially if the work is grounded in critical theories and openly names racism as a driver of inequities. As such, doing this work in communities is bound to upset some people who are committed to maintaining the status quo. The NCCB experienced this firsthand in 2020 when a white supremacist with a criminal history of illegal possession of weapons and assault hacked into our electronic communications system and threatened the NCCB with racist remarks and violence. This came on the heels of George Floyd’s murder and a national reckoning with racism and coincided with COVID’s disproportionate impact on the Black community. Several board members were personally affected and there was a need for healing through connection. The NCCB suspended regular meetings and instead engaged in the six-session Critical Dialogue component of the *Community Wise* intervention, designed to raise awareness of racial inequities. In these sessions, individuals shared personal experiences and engaged in candid conversations about privilege, which ultimately helped to reaffirm relationships and shared purpose among the board members. In the summer of 2021, the NCCB’s website was hacked and destroyed. Although these events were unrelated, in both instances members were able to lean into the supports built within the partnering universities and the police to obtain advice and a minimal level of protection. The NCCB engaged in dialogue to support one another emotionally and identify strategies to protect their members. This included changing passwords and communication strategies. Having a plan to manage trolling and violent attacks is critical to protecting partners and securing confidentiality.

### Collaboration and Capacity Building

#### The North Star and strategic collaboration.

Having a North Star means having a stable and consistent purpose and a future that community members are collectively moving towards. There is a natural tension between trying to include different perspectives while remaining true to the collective core values. We found that having a “north star” (a value or goal that is shared by all collaborators) is critical in guiding the selections of the right partners. For instance, at the beginning of the NCCB, Lili hoped to include law enforcement in the board’s membership. The NCCB spent hours discussing the benefits and challenges of including law enforcement given the historical mistrust between police and community members, the power differential, and the goal to advocate for the rights of marginalized individuals. Moreover, many of the research participants and some board members could be actively using illegal drugs and involved in criminalized activities. The NCCB questioned how to protect their privacy while also including law enforcement in the research process, and ultimately decided to include parole officers and retired members of the police who could share the perspectives of law enforcement with the NCCB and connect the board with key players from law enforcement as needed.

The NCCB learned another important lesson about choosing the right CBPR partners when they launched one study in a partnering CBO whose executive director embraced harm reduction, but the CBO’s mid-level management felt strongly that the abstinence model was the only acceptable approach. Since the NCCB were bringing research participants who were actively using drugs into the CBO for intervention session groups, managers found ways to sabotage the project by blocking access to facilities and resources for people affiliated with the study. The NCCB eventually had to end that relationship and work with another agency. This switch created unnecessary delays to the project and compromised the final sample size and research outcomes. The experience revealed communication gaps between the CBO’s middle and upper management levels that were not initially apparent to the NCCB and prompted the group to think about ways to more thoroughly vet potential partnering CBOs.

#### Functional diversity and the integration of experiential and scientific knowledge in capacity building.

Functional diversity helps to ensure that community members from an array of educational and training backgrounds are collaborators. Our experience highlights the benefits of merging experiential and scientific knowledge to enable mutual learning. Non-research community members mastered theoretical and methodological concepts, measures, and techniques, while researchers gained an in-depth understanding of local service organizations through community connections. This capacity building enhanced community members’ potential for future study involvement. As a result of this process, in 2019, one of our partnering CBOs adopted the CBPR model by incorporating the NCCB as a resource and expanding their research arm. The knowledge generated from this project benefitted all partners, contributing to an evidence-based intervention to reduce substance use. Community members acquired valuable job skills and experience, and researchers gathered data crucial for future studies.

#### Board structure and relationships.

Board members noted that having organized and reliable leadership with funding to support at least some staff is critical to successful collaboration. Someone must set meeting agendas, take meeting notes, make sure that people are doing the work they agreed to do in a timely manner, and bring the dialogue back on track if it goes off topic. Board members are busy people and CBPR work is often done outside of one’s full-time job. It is important to make sure members are not overburdened, that the workload is distributed equitably, and that people’s time is compensated as much as possible. New members should be trained and able to count on another board member willing to serve as a mentor. To survive more than 14 years, the NCCB had to be flexible and able to adapt to environmental changes. Ongoing evaluation was critical to help us identify gaps and issues that needed to be addressed. The use of technology from our inception was critical when Lili moved to Illinois and we had to hold remote meetings and share documents electronically before the widespread adoption of platforms like Box, Skype, and Zoom. Additionally, having healthy relationships and helping all community members feel safe influences the quality of the collaborative output.

At the heart of developing the NCCB was a team of experts committed to addressing health inequities in Newark, New Jersey. As discussed, several founding members are still current and active on the board. They possess valuable insights into the historical decisions and the progress made in the communities served. This dedication not only augments the interpersonal relationships between board members, but it also fortifies research approaches. The trust built among these experts encourages constructive feedback in the NCCB and with their outside projects. Teri emphasizes:

We check on each other. We know what’s going on, and I think that’s really important. You know, moving forward, because, as Lili’s expanding to the Midwest, there are things that we can, you know, where we can maybe give her a little bit of help here and there, or give her an opinion — Wait a minute. You remember when we did this here and how it worked — so that those same mistakes are not made. So, for the core group of us who have been around forever, I think that’s one of our really big strengths for this group.

Teri’s insight elucidates how the board is a collective of experts willing to challenge each other’s perspectives, leveraging the trust built over the last decade, to minimize errors that may reproduce harm in the community. This type of synergy encourages robust and rigorous approaches to conducting CBPR. There is an entire team holding researchers accountable for how to address concerns in the community.

Lili furthers solidifies this sentiment by her testimony that acknowledges the reciprocated respect by all board members,

I think that we are very committed people. We were very innovative. We were cutting-edge thinkers and very committed to making what I call real-world impact. We weren’t doing this because we were trying to advance our careers, and I’m speaking about every single board member.

Emerging from this complicated community-academic partnership process are often novel, context specific, and participatory research approaches focused on a community prioritized issue.

#### Challenges as learning opportunities.

We encountered several challenges, each providing a lesson for future CBPR studies. Understanding institutional policies to anticipate potential problems was key. Hiring CCB members through the CBO rather than the university brought additional funding and expedited hiring processes. Direct funding to CBOs and careful research of potential partner CBOs can ensure compatibility and capacity. Being transparent, sharing the budget, brainstorming solutions, implementing ongoing training and quality control, and hiring CBO staff members were vital practices. CBPR projects should incorporate self-evaluation measures to assess the collaborative process and power sharing, identifying areas for improvement. Recognizing and respecting both scientific and experiential knowledge was critical, as was understanding the situational and relational nature of power. We acknowledged that racism permeates all settings in modern society and engaged in ongoing honest reflection, dialogue, and action to address this pervasive issue within the board, the organizations we partnered with, and the community at large.

CBPR principles are useful in guiding the way that community partners and scientists build knowledge that integrates experiential and scientific expertise. However, the NCCB found that adopting and implementing CBPR principles is messy, complex, time consuming, challenging at times, and ever changing. Yet, the benefits we reaped included the development of enduring friendships and collaborations based on mutual trust, innovative solutions with real-world applications, and a more inclusive process in the search for social justice.

## Conclusion

This paper explored the formation and operation of the Newark Community Collaborative Board (NCCB) and its incorporation of CBPR as a promising paradigm for involving community in efforts to redress health inequities. Using the NCCB as a case example for CBPR to address health inequities highlighted novel and creative methods, delineated lessons learned, identified funding strategies and challenges, explicated interview data analytic/meaning-making techniques, and underscored the importance of community voice. There are several challenges when engaging in CBPR (e.g., power dynamics, funding, leadership, relationships) that the NCCB experienced, in common with other community-engaged research partnerships (Drahota et al., 2016; Israel et al., 2018; Muhammad et al., 2015). Some of these challenges can be anticipated. Successfully addressing others may depend on the quality of the relationships between researchers and community members. Our experience demonstrates that community members make effective research partners who are motivated to help their neighbors, capable of mastering research skills, and able to leverage indigenous expertise to help researchers avoid costly missteps while collaboratively developing sustainable interventions.

Notwithstanding our experience and successes, since 2018, the NCCB has gone through an identity crisis. The NCCB spent many meetings discussing who we are and what we do beyond *Community Wise*. Perhaps it was a perfect storm with Lili relinquishing the Chair position and the completion of *Community Wise*, which had been the source of our work together as a Board for so long. These losses left the NCCB without a clear direction into the future. However, the hacking and loss of our website presented an opportunity to reimagine ourselves once again. Reasserting the Board’s commitment to anti-racism, it unanimously decided to become a chapter of the global Campaign Against Racism. Also, the reimagining process led to the opportunity to create a network of CCBs. The NCCB underwent a name change to become the NJ Critical Consciousness Collaborative Board (NJ-3CB), expanding its reach to different regions in the United States. Lili chairs the Illinois and New Jersey 3CBs. Rogério chairs the Michigan 3CB. Alexis chairs the New York 3CB. The network is in its infancy but represents the NCCB’s growth from a local board to a network of community collaborative boards. How it operates is largely yet to be determined. For now, each of these regions operates independently and can pursue their own funding. What unites us is CBPR and our North Star of critical consciousness. The purpose of the collaborative is to have a space where we can share and exchange ideas related to critical consciousness and CBPR. There is potential to apply for a P30 NIH center which could help each collaborative be more cohesive but still maintain their independence. A center could be a vehicle to provide infrastructure for collaboration, funding, and disseminating our work. If we are correct about the three critical components for the NCCB’s longevity being relationships, funding, and having a North Star, then the network of 3CBs should be around for at least another 14 years.

## Figures and Tables

**Table 1.** NCCB Demographic Information

Sample Characteristics (N = 16)	M (SD) or Number
Age	45.27 (6.52)
Race	
Asian	0
Black	8
White	4
Mixed	4
Ethnicity	
Hispanic	3
Not Hispanic	13
Gender	
Female	10
Male	6
Sexual orientation	
Heterosexual	12
LGBTQ+	4
Highest Level of Education	
Graduate degree	6
Undergraduate degree	8
Completed HS or GED	2
Less than high school	0
Employment	
Full time	10
Part time	5
Unemployed	1
Role on the NCCB	
Consumer	2
Community resident	3
Government representative	1
Researcher	4
Service provider	6
